# Dexamethasone-Functionalized PLLA Membranes: Effects of Layer-by-Layer Coating and Electrospinning on Osteogenesis

**DOI:** 10.3390/bioengineering12020130

**Published:** 2025-01-30

**Authors:** Flavia Gonçalves, Roberta Molisani Letomai, Marjory Muraro Gomes, Maria dos Remédios Aguiar Araújo, Yasmin Silva Muniz, Maria Stella Moreira, Leticia Cidreira Boaro

**Affiliations:** 1 Faculdade de Odontologia, Universidade Santo Amaro, Av. Prof. Eneas de Siqueira Neto, 340, São Paulo 04829-300, SP, Brazilroberta.mletomai@gmail.com (R.M.L.); maria.aguiar.araujo23@gmail.com (M.d.R.A.A.); yasminsmuniz1@gmail.com (Y.S.M.); 2 Departamento de Estomatologia, Hospital AC Camargo, São Paulo 01509-010, SP, Brazil; stellam@usp.br; 3 College of Dentistry, University of Saskatchewan, 107 Wiggins Rd., Saskatoon, SK S7N 5E5, Canada

**Keywords:** scaffolds, dexamethasone, bone differentiation, mesenchymal stem cells

## Abstract

The addition of dexamethasone in membranes for guided bone regeneration is promising due to its dual effect: (1) anti-inflammatory action and (2) induction of osteogenesis in host stem cells. Electrospun fiber coating with dexamethasone using the layer-by-layer (LBL) technique offers an interesting alternative for the gradual release of the drug, aiming for enhanced osteodifferentiation activity. This study aimed to develop synthetic poly-L-lactide (PLLA) membranes with dexamethasone incorporated into the fibers or coated on their surface, and to evaluate the drug release rate, as well as the material’s ability to promote proliferation, osteoconduction, and osteodifferentiation of human periodontal ligament stem cells (hPDLSCs). PLLA membranes were produced by electrospinning. Dexamethasone was incorporated using three techniques: (A) electrospinning of a co-solution of PLLA with 2.5 *w*/*w*% dexamethasone; (B) deposition of four layers on the PLLA membrane using alternating solutions of chitosan and heparin/dexamethasone; (C) deposition of 10 layers on the PLLA membrane using the same solutions. hPDLSC proliferation was measured via CCK-8 at 1, 7, 14, and 21 days. Cellular differentiation was assessed by alkaline phosphatase activity (7 days) and alizarin red staining (21 days) in clonogenic and osteogenic media (ODM). Data were analyzed using one or two-way ANOVA and Tukey test. Electrospun membranes with dexamethasone and those with 4 layers showed immediate drug release within 24 h, whereas 10 layers exhibited gradual release over 14 days. Cumulative drug release was higher for electrospun membranes at 1 and 7 days, similar to 10 layers at 14 and 21 days. The 4 LBL membrane promoted lower hPDLSC proliferation compared to the 10 LBL and electrospun membranes at 21 days but showed increased extracellular matrix mineralization in osteogenic media. No significant differences in alkaline phosphatase expression were observed between materials. Therefore, the addition of dexamethasone in 10 layers, combined with heparin, enables gradual drug release. However, lower drug release in the first 24 h by four LBL membranes improved the material’s osteogenesis properties. None of the materials improved the osteodifferentiation in the clonogenic medium.

## 1. Introduction

Drug delivery systems are promising strategies for guiding tissue responses to achieve various outcomes in bone formation, such as cell recruitment, osteogenesis, and angiogenesis, among others [1,2], and they are an important factor to be considered in the bone tissue engineering for large bone defects regeneration [3].

Scaffolds with delivery of dexamethasone molecule are particularly interesting in this context, since dexamethasone influences bone metabolism through several catabolic and anabolic processes, such as induction of osteogenesis [4,5]; differentiation and maturation of osteoblasts [6]; inhibition of bone resorption by osteoclasts [7]; inhibition of osteoblast proliferation [8]; inhibition of osteocalcin expression [9]; stimulation of osteoclast production [10]; and induction of osteoblast apoptosis [11]. A previous study demonstrated that a chitosan–alginate–gelatin scaffold combined with dexamethasone (DEX)-loaded mesoporous silica nanoparticles enhanced osteogenesis in vivo [12], whereas, in contrast, rats treated with injectable dexamethasone showed suppresses osteogenesis in vivo [13].

Given the wide range of possible responses to dexamethasone, understanding the mechanism of its incorporation into the scaffolds and its release kinetics is crucial for determining the optimal dose, exposure duration, and, consequently, the type of response achieved [14]. Electrospinning is a widely used technique for creating drug-loaded membranes [15]. When the drug is in co-solution with the polymer, part of the drug is deposited on the surface of the fibers and has a faster release, while the rest is incorporated within the fibers. Its release depends on the drug’s ability to diffuse through the polymer chains and the polymer degradation rate [15]. Another technique for fabricating drug-loaded membranes is the layer-by-layer (LBL) method, which involves the alternating deposition of cationic and anionic polyelectrolytes, stabilized by electrostatic interactions, forming layers that encapsulate the drug of interest [16]. Drugs in the more superficial layers are released more quickly, while those in deeper layers take longer to diffuse and be released. The release kinetics depend on the size and charge of the drug [17].

The objectives of this study were (1) to synthesize poly-L-lactic acid membranes with incorporated dexamethasone using the electrospinning co-solution technique and the layer-by-layer technique with 4 or 10 layers; (2) to evaluate the drug release profile in the different materials; (3) to assess the effect of the dexamethasone-loaded membranes on the proliferation, osteogenesis, and osteoinduction of human mesenchymal stem cells derived from periodontal ligament. The hypothesis of the study is that a more gradual drug release will enhance osteoinduction, osteogenesis, and cellular proliferation.

## 2. Materials and Methods

Four types of membranes were fabricated, as follows: (1) electrospun PLLA membranes; (2) electrospun membranes from a co-solution of PLLA/dexamethasone; (3) electrospun PLLA membranes coated with 4 layers of layer-by-layer (LBL) heparin + dexamethasone/chitosan; and (4) electrospun PLLA membranes coated with 10 layers of LBL heparin + dexamethasone/chitosan (Table 1).

### 2.1. Synthesis of Membranes by Electrospinning

For the synthesis of the PLLA polymeric membranes, 5 *w*/*v*% PLLA was dissolved in chloroform. After dissolution, dimethylformamide was added in a ratio chloroform: dimethylformamide of 9:1, and the solution was electrospun under the following parameters: injection rate of 3 mL/h, needle-to-collector distance of 12 cm, and voltage of 20 kV.

The PLLA electrospun membranes served as the control group and as the substrate for incorporating dexamethasone onto the fiber surface using the layer-by-layer technique.

The dexamethasone was added to the PLLA solution to create a co-solution containing 5 *w*/*v*% PLLA and 2.5 *w*/*w*% dexamethasone (relative to the polymer mass), dissolved in a 9:1 chloroform/di-methylformamide mixture. This solution was then electrospun using the same parameters as previously described.

### 2.2. Incorporation of Dexamethasone by the Layer-by-Layer Technique

**Figure 1:**
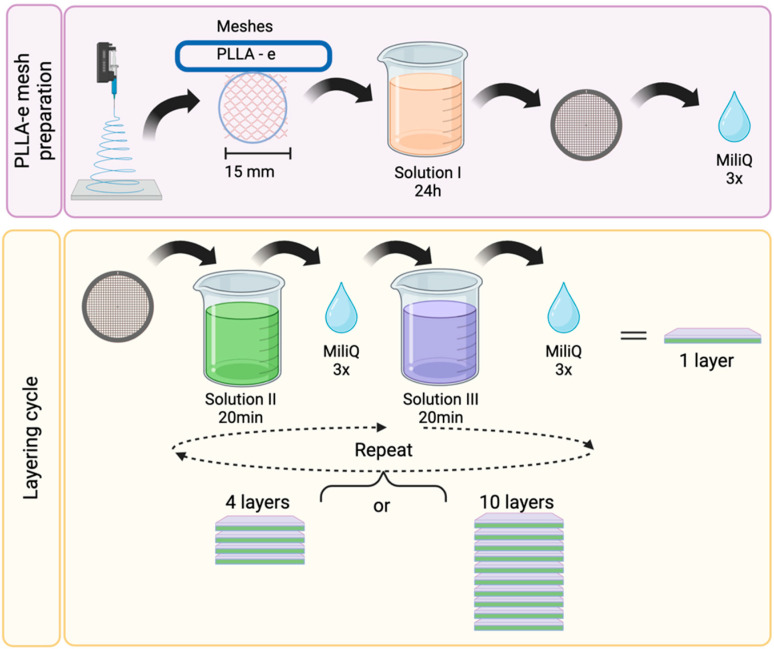
Layering technique. Schematic illustration of the layering technique. Solution I: 2.5 mg/mL branched polyethylenimine. Solution II: 1 mg/L heparin and 0.15 M sodium chloride, containing 0.05 mg/mL of diluted dexamethasone. Solution III: 1 mg/mL chitosan and 0.15 M sodium chloride in 2 *v*/*v*% acetic acid. The successive immersion procedure Solution II and Solution II was repeated to obtain the desired number of layers (4 and 10 layers). Figure created in https://BioRender.com (agreement number #AN27SYH82M).

In this technique, dexamethasone molecules were incorporated onto the PLLA fibers of the electrospun meshes (Figure 1). Initially, the meshes were cut into 15 mm diameter discs and immersed in a solution containing 2.5 mg/mL branched polyethylenimine for 24 h (Solution I). For the first biomolecule layer, the membranes were washed three times with MilliQ water and immersed for 20 min in a solution of 1 mg/L heparin and 0.15 M sodium chloride, containing 0.05 mg/mL of diluted dexamethasone (Solution II). The meshes were washed again and immersed for 20 min in a solution of 1 mg/mL chitosan and 0.15 M sodium chloride in 2 *v*/*v*% acetic acid (Solution III). The samples were washed once more, and the successive immersion procedure in heparin and dexamethasone/chitosan solutions was repeated to obtain the desired number of layers (4 and 10 layers).

### 2.3. Membranes Characterization

Samples were dried in a vacuum desiccator, coated with 6 nm of gold, and analyzed in a scanning electron microscope (SEM) (FEG 7401F, Jeol, Tokyo, Japan) at ×10,000 magnification for morphology analysis. The average fiber diameter was measured using Image J software 1.53e (National Institutes of Health, Bethesda, MA, USA) with 50 measurements per material.

For the analysis of dexamethasone release, the meshes (Ø = 15 mm) were sterilized under ultraviolet light for 15 min on each side and immersed in 6 mL of phosphate buffer saline (PBS), pH 7, containing 0.2 *v*/*v*% Tween-20. Aliquots of 1 mL were removed at 1, 7, 14, and 21 days, and the same volume of PBS and Tween-20 solution was added to the samples to maintain volume. Dexamethasone quantification was performed by UV–vis spectroscopy at an absorbance of 242 nm, compared to a previously constructed calibration curve.

### 2.4. Isolation of Periodontal Ligament Stem Cells

Primary cultures of periodontal ligament cells were obtained from fragments of periodontal ligament of human permanent teeth using the explant technique, as previously described [18], after submission and approval by the Research Ethics Committee and with informed consent from the patients. Briefly, third molars teeth with surgical indication were extracted and periodontal ligament human tissue was removed from the middle third of the root, using a scalpel blade. The tissue was washed abundantly with PBS and cut in small fragments (explants), which were transferred to a Petri dish and immersed in 0.25% trypsin solution for 5 min at 37 °C. The explants were culture in Dulbecco’s Modified Eagle Medium/Ham’s F-12 medium (Vitrocell, Campinas, Brazil), supplemented with 10 *v*/*v*% fetal bovine serum (Vitrocell), and 1 *v*/*v*% penicillin/erythromycin (10,000 U/mL/10,000 µg/mL) until the cells migrate out the explants. The cells from the fragments were detached using trypsin and plated on new culture flasks. The cultures were kept in semi-confluence until use or freezing, to avoid differentiation.

### 2.5. Cell Culture

The membranes were sterilized under ultraviolet light for 15 min on each side. Cells were cultured on the membranes in clonogenic medium or osteodifferentiation medium (ODM) depending on the test to be performed. Clonogenic medium consists of Dulbecco’s Modified Eagle Medium (DMEM) with high glucose, 10 *v*/*v*% fetal bovine serum, and 1 *v*/*v*% penicillin/erythromycin (10,000 U/mL/10,000 µg/mL). ODM consists of a clonogenic medium supplemented with 50 µg/mL ascorbic acid and 10 mM β-glycerophosphate.

### 2.6. Cell Proliferation Assay

The membranes (Ø 15 mm) were plated with 20,000 cells per well, cultured in clonogenic medium for cell adhesion and proliferation analysis by the Alamar Blue assay. The culture medium was changed every two days. After 1, 7, 14, and 21 days, cell viability of the periodontal ligament stem cells (PLSCs) adherent to the membranes was assessed using the Cell Counting Kit-8 (CCK-8, Sigma-Aldrich, São Paulo, Brazil). The membranes were transferred to a new plate, and a 10 *v*/*v*% CCK-8 solution in serum-free culture medium was added to the wells, followed by incubation for 3 h. The supernatant was collected in 96-well plates, and absorbance at 450 nm was measured using a microplate reader.

### 2.7. Alkaline Phosphatase Assay

For alkaline phosphatase measurement, 1 × 10^5^ cell/well were plated onto the membranes and cultured for 7 days in ODM or clonogenic medium. The membranes were washed with PBS, transferred to a new plate, and immersed for 15 min in a solution of 1 mg/mL p-nitrophenyl phosphate in 0.05 M glycine and 2.2 mM MgCl_2_, pH 10.5. The alkaline phosphatase enzyme cleaves p-nitrophenyl phosphate into p-nitrophenol, forming a yellow-colored solution. The reaction was stopped by transferring the solution to a tube with NaOH in the same volume. Absorbance was measured at 405 nm.

### 2.8. Alizarin Red Assay

In 24-well plates, 1 × 10^5^ cells/well were plated onto the membranes and cultured in ODM or clonogenic medium for 21 days. After 21 days, the meshes were washed with PBS, fixed in 10 *v*/*v*% formalin for 10 min, and immersed for 3 min in an aqueous solution containing 1 *w*/*v*% alizarin red and 2 *v*/*v*% absolute ethanol. The membranes were washed thoroughly and desorbed in 10 *w*/*v*% cetylpyridinium chloride solution for 1 h. Absorbance of the desorbed solution was measured at 570 nm, using a spectrophotometer.

### 2.9. Statistical Analysis

Respecting the requirements for normality and homoscedasticity, the data were analyzed using one-way ANOVA (ALP and Alizarin red assay), two-way ANOVA (dexamethasone release and proliferation assay), both with Tukey’s test for comparison of means, and Kruskal–Wallis’s test (mean fiber diameter) with Student–Newman–Keuls test for mean comparisons. All tests were performed with a global significance level of 95% (α = 0.05).

## 3. Results

### 3.1. Membranes Characterization

**Figure 2:**
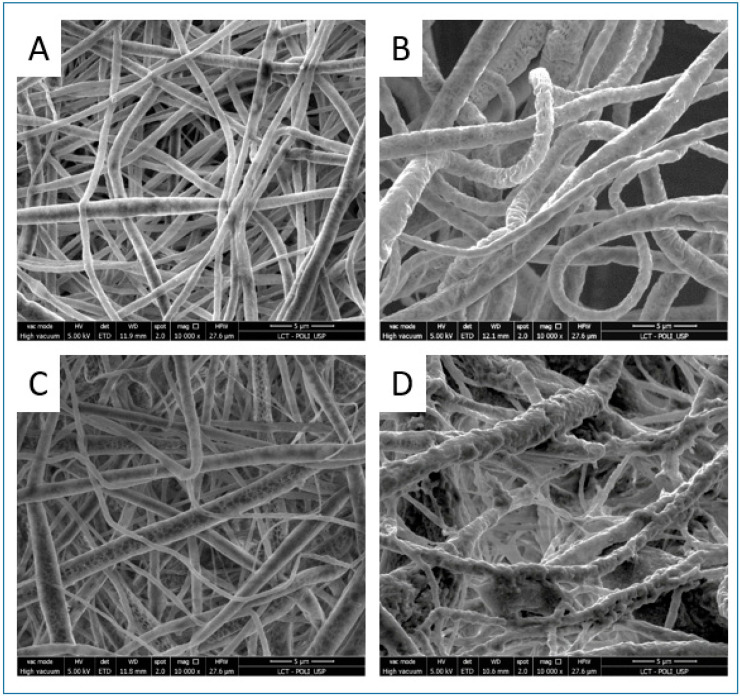
Scaffolds—scanning electron microscopy. Scanning electron microscopy (SEM) images with 10,000× magnification of (**A**) PLLA membranes; (**B**) electrospun membranes from a co-solution of PLLA/dexamethasone; (**C**) electrospun PLLA membranes coated with 4 layers of layer-by-layer (LBL) heparin + dexamethasone/chitosan; (**D**) electrospun PLLA membranes coated with 10 layers of LBL heparin + dexamethasone/chitosan.

The membranes obtained by electrospinning a PLLA solution alone and those coated with 4 or 10 layers of layer-by-layer (LBL) deposition exhibited similar average fiber diameters (0.6 ± 0.3 µm), which were smaller compared to the fibers produced by the co-solution of PLLA and dexamethasone (1.4 ± 0.5 µm). The deposition of 4 LBL layers of heparin and dexamethasone/chitosan did not alter the morphology of the mat, whereas the addition of 10 layers resulted in the formation of plaques between the fibers (Figure 2).

### 3.2. Dexamethasone Cumulative Release

**Figure 3:**
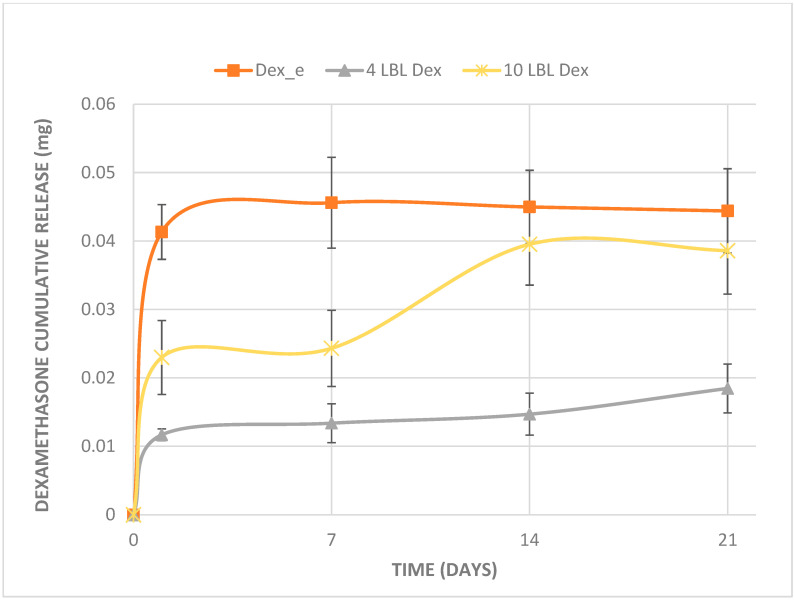
Dexamethasone cumulative release. Mean and standard deviation of cumulative dexamethasone release (mg) over time in membranes obtained by electrospinning of co-solution and the layer-by-layer technique.

The total release profiles differed over the 21-day period among the materials (Figure 3). The electrospun mat exhibited a rapid burst release on day 1 and statically presented a higher dexamethasone release than the other materials at 1 and 7 days. The four LBL material demonstrated the lowest release of dexamethasone throughout the 21-day period, with an initial burst on day 1 and no significant difference through the 21 days. Statistically, it showed lower cumulative release than the other materials at days 14 and 21. The 10 LBL material displayed an intermediate release profile, showing low release at 1 and 7 days, similar to the 4 LBL material. However, at 14 and 21 days, the release increased, reaching dexamethasone release levels comparable to the electrospun mat.

### 3.3. Cell Proliferation Assay

**Figure 4:**
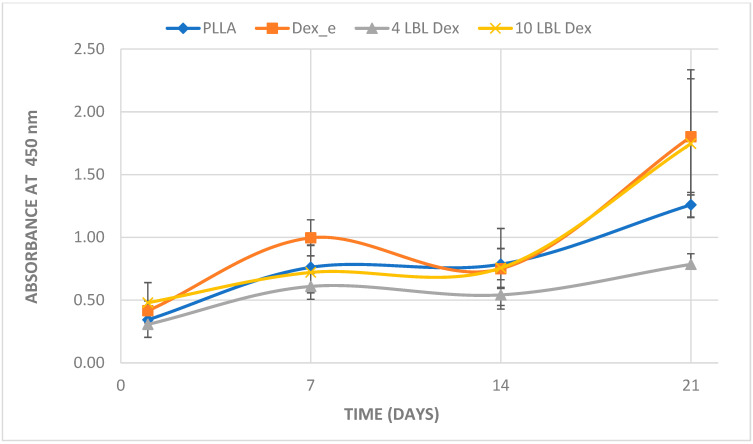
Proliferation assay (CCK-8 kit). Mean and standard deviation of absorbance at 450 nm related to the proliferation assay of hPDLSC over the membranes, as a function of time, using the CCK-8 kit.

All materials supported hPDLSC adhesion and proliferation, with similar proliferation observed on day 1. However, the 4 LBL material exhibited lower proliferation compared to the electrospun dexamethasone material at 7 days, and also at 21 days, when compared to both the electrospun dexamethasone and 10 LBL materials. The other materials showed similar proliferation rates at the time points analyzed (Figure 4).

### 3.4. Alkaline Phosphatase Assay

**Figure 5:**
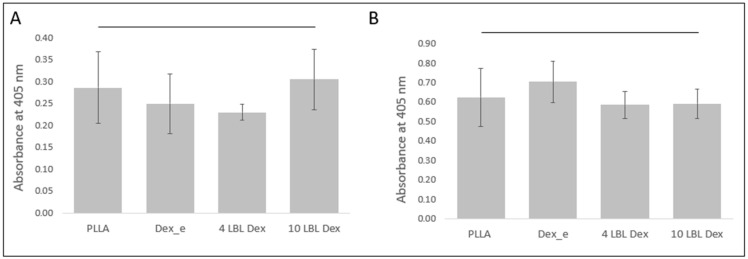
Alkaline phosphatase assay. Mean and standard deviation of absorbance at 405 nm related to the alkaline phosphatase assay at 7 days of culture of hPDLSCs in (**A**) clonogenic medium and (**B**) osteogenic medium.

The alkaline phosphatase (ALP) assay for hPDLSCs cultured on the materials revealed no significant differences between the materials, regardless of whether the cells were cultured in clonogenic medium (Figure 5A) or osteogenic differentiation medium (Figure 5B). However, higher ALP activity was observed in cultures grown in the osteogenic medium.

### 3.5. Alizarin Red Assay

**Figure 6:**
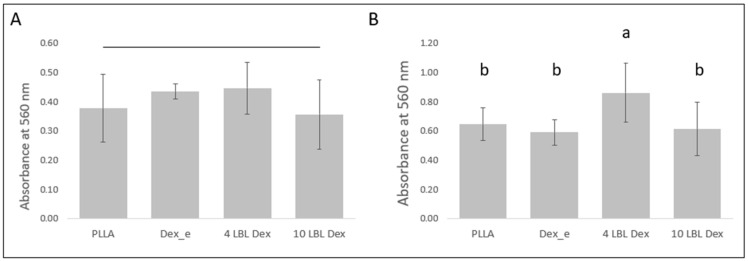
Alizarin red assay. Mean and standard deviation of absorbance at 570 nm related to the Alizarin red assay at 21 days of culture of hPDLSCs in (**A**) clonogenic medium and (**B**) osteogenic medium. Similar letters indicate absence of statistically significant difference.

In the clonogenic medium, no significant differences in extracellular matrix mineralization were observed between the materials (Figure 6A). In osteogenic medium, hPDLSCs cultured on membranes with dexamethasone incorporated in four layers of LBL exhibited greater extracellular matrix mineralization, as assessed by the Alizarin red assay, compared to the other materials (Figure 6B).

## 4. Discussion

This study demonstrated that both the incorporation of dexamethasone into poly-l-lactic acid (PLLA) membranes by electrospinning of a co-solution and the deposition of the drug onto the polymeric fiber surface using the layer-by-layer (LBL) technique were feasible and exhibited distinct drug release profiles. While the incorporation of dexamethasone via electrospinning of a co-solution has been previously described in the literature [19,20], its incorporation using the layer-by-layer technique is novel and was validated in this study. Membranes with 10 layers of LBL deposition altered the morphology of the mats, creating regions of fiber interconnection, which was not observed in membranes with 4 LBL layers. On the other hand, the presence of dexamethasone in the co-solution increased the fiber diameter, likely due to the molecular structure of dexamethasone, which contains hydroxyl and carbonyl groups that affect the solution’s viscosity, leading to an increase in the average fiber diameter. Indeed, a study on electrospun PDLA membranes demonstrated that adding dexamethasone (1–10 *w*/*w*% by weight) resulted in a slight increase in the average fiber diameter [21].

The number of LBL layers is inversely related to the drug release rate, the greater the number of layers, the higher the interaction between the drug and the electrolytes, resulting in slower drug release [17]. This was observed in the 10-layer material, which took longer than 14 days for the drug release to increase significantly, in contrast to the 4-layer material, which released most part of the drug in just 1 day. Regarding dexamethasone release in mats electrospun by co-solution, as it has low molecular weight, and is a hydrophobic molecule, its diffusion through the hydrophobic PLLA fibers is facilitated, leading to its release on the first day, with concentrations higher than those obtained for the other materials.

Dexamethasone can either stimulate or inhibit cell proliferation depending on concentration and exposure time [8,20]. All materials promoted similar cell adhesion on day 1, suggesting no advantage of dexamethasone at this point, possibly due to the short exposure time. On days 7 and 21, the material with the lowest dexamethasone release (four-layer LBL) exhibited lower cell proliferation compared to the material with the highest dexamethasone release, the electrospun membrane. The same pattern was observed on day 21, where the 4-layer LBL material exhibited lower proliferation than both the electrospun membrane and the 10-layer LBL membrane.

Finally, the effect of dexamethasone release profiles on osteodifferentiation and osteogenesis was evaluated using alkaline phosphatase and Alizarin red assays. In clonogenic medium, all cellular differentiation was attributed to the material itself, as none of the materials promoted osteodifferentiation either in the early stages (as shown by alkaline phosphatase assay) or in the late stages (as shown by Alizarin red assay). Therefore, dexamethasone alone was unable to stimulate osteodifferentiation of hPDLSCs. Although some studies report dexamethasone’s ability to promote osteogenesis, they typically associate it with other factors such as ascorbic acid, BMP-2, or vitamin D, maintaining an osteogenic medium that supports dexamethasone’s effects [5,22,23,24]. Other studies also indicate that high doses of dexamethasone inhibit osteogenesis even in the osteogenic medium, inducing adipogenesis, and promoting cellular apoptosis [25]. Thus, the osteodifferentiation capacity of dexamethasone is complex, depending on dose and interaction with other factors.

When analyzing the effect of these materials in osteogenic medium, it was observed that while dexamethasone release profiles did not affect the early stages of cellular differentiation (as measured by alkaline phosphatase synthesis), they did influence extracellular matrix mineralization. The material with four LBL deposition, which released a low dose of dexamethasone with a significant release on day 1, showed the best results in terms of matrix mineralization. The effect of dexamethasone on alkaline phosphatase expression has been reported in several studies [26,27,28]. It appears that cell lineage, the degree of cellular differentiation at the time of dexamethasone addition, and the concentration used are critical factors for this process [28,29]. Higher doses (above 10^−8^ M) lead to downregulation of the PI3K/Akt pathway phosphorylation, reducing alkaline phosphatase expression in neonatal rat calvaria cells or rat bone marrow cells [26,29]. Doses of 10^−7^ M, on the other hand, increased alkaline phosphatase expression in studies using chicken embryo calvaria cells [30] and human bone marrow stem cells [27]. However, in the present study, the doses used do not alter the alkaline phosphatase in relation to the control group of PLLA membranes.

In other ways, improvements in osteogenesis were observed in this study during the late-stage maturation of bone cells, as indicated by extracellular matrix mineralization in membranes with four LBL deposition. Higher doses of dexamethasone released on day 1 from co-electrospun membranes or on day 14 from membranes with 10 LBL deposition may have resulted in feedback inhibition of the osteogenesis process. In fact, one study has shown that human bone cells express leptin receptors, which are upregulated by dexamethasone in a dose-dependent manner [9]. Thus, cyclically, the presence of leptin downregulates osteocalcin expression, decreasing bone mineralization also in a dose-dependent manner [9].

It should be highlighted that this is an in vitro study, and other factors may affect the materials performance in vivo; also, it was carried with one cell lineage and others lineage could respond in a different way. Considering these limitations, it can be concluded that the synthesis of bioactive membranes with dexamethasone release is feasible and they perform different dexamethasone release kinetics over time; cell proliferation increases with dexamethasone dose and exposure duration; and dexamethasone, under the evaluated conditions, is not able to induce osteodifferentiation but does enhance extracellular matrix mineralization in osteogenic medium when released at low doses over time.

Future studies could address different concentrations and designs of dexamethasone load in membranes covered with layer-by-layer technique. In the present study, 4 and 10 layers were tested, with dexamethasone present in all the layers, in order to reach a fast and slow dexamethasone release, respectively. However, other arrangements can reach different cell responses. Also, future studies should evaluate the influence of the best profiles of dexamethasone release in vivo, since the drug has known effects in both osteoblasts and osteoclasts.

## Figures and Tables

**Table 1:** Description of control and experimental groups evaluated.

Groups	Bioactive Molecule	Incorporation Technique	Concentration/Number of Layers
PLLA	-	-	-
Dex_e	Dexamethasone	Electrospinning	0.175 mg/mL
4 LBL_dex	Dexamethasone	Layer-by-layer	0.05 mg/mL/ 4 layers
10 LBL_dex	Dexamethasone	Layer-by-layer	0.05 mg/mL/ 10 layers

## Data Availability

Dataset available on request from the authors.
